# Soft Tissue Augmentation at Single Implants With Collagen Matrix or Connective Tissue Graft: 7.5‐Year Follow‐Up of a RCT

**DOI:** 10.1111/cid.70146

**Published:** 2026-04-13

**Authors:** Margherita G. Liguori, Franz J. Strauss, Thomas J. W. Gasser, Ronald E. Jung, Nadja Naenni, Daniel S. Thoma

**Affiliations:** ^1^ Clinic of Reconstructive Dentistry, University of Zurich Zurich Switzerland; ^2^ Department of Life, Health and Environmental Sciences University of L'Aquila L'Aquila Italy; ^3^ Universidad Autonoma de Chile Santiago Chile; ^4^ Private Practice Basel Switzerland; ^5^ Department of Periodontology Research Institute for Periodontal Regeneration, Yonsei University College of Dentistry Seoul South Korea

**Keywords:** collagen matrix, connective tissue, dental implants, esthetics, patient‐reported outcome measures, peri‐implant mucosa, soft‐tissue augmentation

## Abstract

**Aim:**

To compare up to 7.5‐year clinical and patient‐reported outcomes of implant sites previously augmented using a volume‐stable collagen matrix (VCMX) or connective tissue graft (SCTG) in the esthetic zone.

**Methods:**

The original randomized controlled trial (RCT) enrolled 20 patients who received soft tissue augmentation with VCMX or SCTG at single implant sites. Clinical assessments and standardized measurements were performed at baseline after crown insertion (BL) and at 6 months, 1, 3, 5, and 7.5 years. The primary outcome was mucosal thickness. Secondary outcomes included marginal bone levels, probing depth, bleeding on probing, plaque index, pink esthetic score (PES), OHIP‐14, and buccal profilometric changes. Group comparisons were performed using mixed‐effects and generalized estimating equation (GEE) models, which account for within‐patient correlations due to repeated measurements and allow inclusion of all available data without requiring imputation for missing observations.

**Results:**

Of the 20 originally recruited patients, 12 were available for re‐examination at 7.5 years (five in the SCTG group and seven in the VCMX group). Mucosal thickness at crown delivery ranged from 2 to 4 mm across the study population. Adjusted mean differences (VCMX–SCTG) in mucosal thickness were +0.70 mm at BL, −0.02 mm at 6 months, +0.1 mm at 1 year, +0.1 mm at 3 years, +0.1 mm at 5 years and −0.1 mm at 7.5 years. None of these differences reached statistical significance (*p* > 0.05). For all secondary outcomes, no statistically significant inter‐group differences were detected (*p* > 0.05) except for BOP at 5 years follow‐up.

**Conclusion:**

Both augmentation approaches achieved stable peri‐implant mucosal dimensions, preserved marginal bone levels, and favorable esthetic and patient‐reported outcomes over 7.5 years. Volume‐stable collagen matrices might be a viable alternative to SCTG for peri‐implant soft‐tissue augmentation; however, adequately powered RCTs are needed to confirm these long‐term observations.

**Trials Registration:**

DRKS00017484.

## 1 Introduction

Soft‐tissue augmentation has evolved into a routine component of implant therapy, as increased mucosal thickness supports long‐term peri‐implant stability and enhances esthetic integration [1, 2, 3]. Depending on the clinical scenario, these interventions may be incorporated before implant placement, performed simultaneously, or scheduled during early healing or after completion of the final restoration [4].

Augmenting mucosal thickness provides several clinical benefits. During early healing, grafting supports the re‐establishment of the natural ridge contour and plays a key role in shaping the final peri‐implant soft‐tissue profile [5]. Over the long term, a thicker mucosa has been associated with shallower probing depths, reduced plaque accumulation, fewer bleeding sites, and more stable marginal bone levels [6, 7]. Adequate soft‐tissue volume also enhances esthetic outcomes by minimizing the risk of abutment shine‐through when mucosal thickness is below 2 mm [8, 9]. Furthermore, soft‐tissue augmentation has been linked to improved long‐term stability of the mucosal margin [4, 10].

Subepithelial connective tissue grafts (SCTGs) remain the gold standard for mucosal thickening due to their predictable biological performance and favorable esthetic outcomes [11, 12, 13, 14]. Nevertheless, the advantages of SCTG are counterbalanced by substantial drawbacks. Harvesting requires a second surgical site, is subject to anatomical variability, and is frequently associated with patient discomfort [15, 16] and delayed healing at the donor area [17, 18]. In contemporary practice, where patient‐reported outcomes (PROs) are central to evaluating treatment success and guiding decision‐making, such disadvantages are highly relevant [19, 20, 21, 22, 23].

In response to these limitations, a variety of soft tissue substitutes have been introduced [20, 22, 24, 25]. Early clinical investigations suggest that these materials can provide clinically relevant increases in keratinized tissue width and mucosal thickness, often performing comparably to SCTG while reducing patient morbidity [15, 24, 26].

Despite these promising findings, data on their long‐term performance remain scarce. Most available studies are restricted to short‐term and mid‐term follow‐up, typically within 3 years and only one with observations at 5 years [27, 28, 29, 30, 31, 32]. This gap is clinically relevant, as previous studies have reported progressive shrinkage of substituted tissues over time [31, 32, 33]. Understanding whether soft tissue substitutes can maintain peri‐implant stability and esthetic outcomes in the long term is essential for informed, evidence‐based treatment planning.

The present study, therefore, reports 7.5‐year outcomes at implant sites augmented with either a soft tissue substitute or an autogenous connective tissue graft in the anterior region, evaluating mucosal thickness, contour stability, peri‐implant health, esthetics, and PROs.

## 2 Materials and Methods

### 2.1 Study Design

This investigation represents the 7.5‐year follow‐up of a randomized controlled clinical trial (RCT) [34] conducted at the Clinic of Reconstructive Dentistry, Center of Dental Medicine, University of Zürich, Switzerland. The original trial was designed and reported in accordance with the CONSORT statement for randomized trials and the principles of the Declaration of Helsinki. Ethical approval was granted by the local ethics committee (Kantonale Ethikkommission Zürich; KEK‐ZH‐Nr 2011‐0408), and the long‐term extension was approved under KEK‐ZH‐Nr 2012‐0226. The study was registered in the German Clinical Trials Register (DRKS00017484). All participants provided written informed consent before enrolment.

### 2.2 Inclusion Criteria

Specific inclusion criteria involved the following:

1. Patients previously enrolled in a RCT [34] and re‐examined annually up to 7.5 years post insertion of final restorations.
2. Final restoration inserted at the implant site.
3. Ability to fully understand the nature of the proposed non‐interventional long‐term follow‐up study and the ability to sign the informed consent form.

### 2.3 Exclusion Criteria

1. Newly developed disease interfering with soft tissue regeneration (e.g., diabetes).
2. Peri‐implant infection (not related to previously performed soft tissue regeneration) following the insertion of the final reconstruction.
3. Second soft tissue augmentation since completion of study.
4. Severe trauma to implant site.
5. Orthodontic treatment in the same quadrant.
6. Patients not willing to participate in the 7.5‐year follow‐up examination.

### 2.4 Clinical Procedures

All 20 participants enrolled in the original RCT underwent a soft tissue augmentation procedure at a single‐tooth implant site that had healed under submerged conditions. After local anesthesia, incisions were made around the adjacent teeth to access the edentulous area. A horizontal incision was then extended across the crest, either straight or slightly shifted to the palatal/lingual side, connecting the mesial and distal line angles of the neighboring teeth. On the buccal aspect, a split‐thickness flap was carefully elevated to create a pouch that exceeded the dimensions required to accommodate the planned graft.

At this point, randomization was revealed by opening a sealed envelope assigning the patient to one of the following interventions:

– *VCMX*: augmentation with a cross‐linked, volume‐stable collagen matrix (Geistlich Fibro‐Gide, Geistlich Pharma AG, Wolhusen, Switzerland).
– *SCTG*: augmentation with an autogenous subepithelial connective tissue graft harvested from the palate using a single‐incision technique.

In the VCMX group, the block (25 × 25 × 8 mm) was adapted and shaped to conform to the morphology of the recipient site. The graft (VCMX or SCTG) was positioned within the pouch and stabilized to the palatal flap with sutures. Primary closure without tension was obtained using a combination of horizontal mattress and interrupted sutures (Gore‐Tex 5–0; W.L. Gore & Associates, Flagstaff, AZ, USA).

Postoperative care included rinsing twice daily with 0.2% chlorhexidine solution (Hibitane; AstraZeneca), systemic anti‐inflammatory medication (Ponstan; Parke‐Davis), and systemic antibiotics (amoxicillin 2.25 g/day; Sandoz) for 7 days. To avoid trauma to the augmented area, temporary removable partial dentures were relined accordingly. Sutures were removed 7–10 days postoperatively. After a healing period of 3 months, implant uncovery was performed by abutment connection using a minimally invasive U‐shaped approach. Fixed provisional restorations were placed and maintained to allow soft‐tissue conditioning and contour maturation, followed by delivery of definitive screw‐retained single crowns. Patients were then scheduled for the baseline examination and enrolled in an individualized maintenance program throughout the entire observation period.

### 2.5 Baseline and Follow‐Up Examinations

Baseline (BL) for the long‐term study was defined as 2 weeks after crown delivery. Patients were examined at BL, 6 months (FU‐6M), 1 year (FU‐1), 3 years (FU‐3), and 5 years (FU‐5), as previously reported, and were invited for a 7.5‐year recall visit (FU‐7.5). All examinations were performed by a blinded examiner not involved in the previous RCT and unaware of the therapy the patients had received. The maintenance recalls were tailored according to each patient, ranging from 3 to 6 months.

### 2.6 Outcome Measures

#### 2.6.1 Primary Outcome Measure: Mucosal Thickness

The mucosal thickness was assessed using an endodontic file (K‐File 31/15) inserted 1 mm apical of the mucosal margin on the buccal side. Changes in mucosal thickness over time (BL to FU‐7.5) were considered as primary outcome.

#### 2.6.2 Secondary Outcome Measures

##### 2.6.2.1 Buccal Contour Changes

At each follow‐up visit, impressions of the implant site and the two adjacent teeth were taken using an A‐silicone material. Casts were poured and digitized with a laboratory scanner to obtain stereolithography (STL) files, which were then imported into a digital imaging software program (SMOP, Swissmeda, Zurich, Switzerland). For each case, a trapezoid‐shaped region of interest (ROI) was defined as follows: the coronal border was placed 1 mm apical to the mucosal margin, the apical border corresponded to the mucogingival junction, and the mesial and distal borders were located 1 mm from the neighboring teeth. Due to interindividual anatomical differences, the absolute dimensions of the ROI varied between patients, but once established at the first assessment, the same ROI was applied consistently to all subsequent time points. STL datasets from baseline and the follow‐ups (6 months, 1 year, 3 years, 5 years, and 7.5 years) were superimposed using a best‐fit alignment on the surfaces of the neighboring teeth. The software then calculated the mean surface distance between datasets within the ROI, expressed in millimeters (Figures 2 and 3).

##### 2.6.2.2 Clinical and Periodontal Measurements

Probing depth (PD), the plaque control record (PCR), bleeding on probing (BOP) were assessed at six sites for all implants and the respective two neighboring teeth. The presence of peri‐implant health or disease was assessed according to the report of the 2017 World Workshop on the Classification of Periodontal and Peri‐Implant Diseases and Conditions [7].

##### 2.6.2.3 Diagnosis of Peri‐Implant Conditions

Peri‐implantitis was diagnosed according to the 2017 World Workshop [7]. Peri‐implant mucositis was defined according to the updated ID‐COSM consensus as bleeding (more than one spot on gentle probing) without bone loss beyond initial crestal bone remodeling [35, 36].

##### 2.6.2.4 Radiologic Examination and Assessment of Bone Loss

Standardized periapical radiographs were obtained at all follow‐up visits using the long‐cone paralleling technique with digital sensor holders. Marginal bone levels were assessed by measuring the distance between the implant shoulder and the first bone‐to‐implant contact (fBIC) at the mesial and distal aspects of each implant. The mean of both sites was considered for analysis. Radiographic measurements were calibrated using known implant dimensions (inter‐thread pitch or implant length) to correct for potential radiographic distortion. All measurements were performed by a single examiner who was independent of the surgical and prosthetic procedures. To assess intra‐examiner reliability, measurements were repeated on two separate occasions at least 1 month apart. For the second assessment, radiographs from 10 patients were randomly selected using a computer‐generated sequence (www.randomizer.org).

Intra‐examiner reliability was assessed using the intraclass correlation coefficient (ICC) based on a two‐way mixed‐effects model with absolute agreement. The average ICC was 0.94 for mesial sites and 0.99 for distal sites, indicating excellent agreement.

##### 2.6.2.5 Esthetics

Peri‐implant soft‐tissue esthetics were evaluated by a blinded, calibrated examiner using the PES [37].

##### 2.6.2.6 PROs

PROs were assessed at each follow‐up time point using a standardized and validated patient‐reported outcome measure (PROM), namely oral health impact profile‐G14 (OHIP‐G14).

### 2.7 Randomization Procedure, Concealment of Allocation and Outcome Assessments

To ensure balanced distribution of patients between the two treatment groups, a block randomization scheme was applied. Allocation concealment was maintained by a study monitor who prepared the assignments in opaque, sealed envelopes, which were opened intraoperatively once the recipient site had been prepared. Owing to the nature of the interventions, blinding of the surgeons was not feasible; however, all outcome assessments were carried out by an independent examiner who remained unaware of the treatment allocation and had not participated in the surgical phase.

### 2.8 Sample Size

The original sample size was calculated to assess the noninferiority of VCMX compared with SCTG for changes in soft‐tissue thickness [26]. Assuming a noninferiority margin of 1 mm, an SD of 0.5 mm, and a 30% dropout rate, 10 patients per group were required to achieve 95% power at a one‐sided alpha of 0.025.

### 2.9 Statistical Analysis

Descriptive statistics (mean, SD, median, Q1, and Q3) were calculated for all metric variables. To compare buccal mucosal thickness (primary outcome), a linear mixed‐effects model was fitted to account for within‐subject correlations arising from repeated measurements. Fixed effects included treatment group, time, and their interaction, allowing estimation of treatment effects at each time point. Patients were modeled as random effects.

Missing outcome data were handled with mixed‐effects model, which incorporates all available observations without requiring explicit imputation and provides valid estimates under a missing‐at‐random assumption. As a result, all participants with at least one post‐baseline measurement contributed to the long‐term analysis. Model assumptions were assessed visually using residual diagnostics (Q–Q plots and histograms). When assumptions were not met, nonparametric general linear models with generalized estimating equations (GEEs) were applied. Secondary outcomes including marginal bone levels (MBL), probing depth (PD), bleeding on probing (BOP), plaque index (PI), PES, OHIP‐14, and volumetric changes were evaluated per protocol and compared using mixed‐effects or GEE models as appropriate. The significance level (*α*) was set at 5%, and all tests and *p*‐values were two‐sided. All analyses were conducted using Stata v19.5 (StataCorp, College Station, TX).

## 3 Results

### 3.1 Patients

**FIGURE 1:**
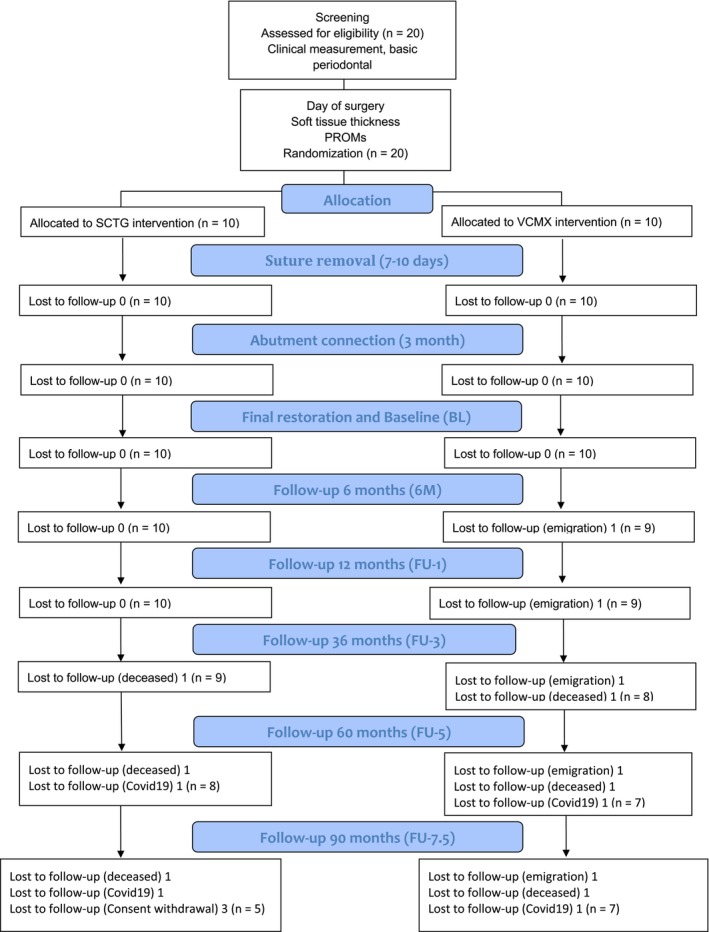
CONSORT flow diagram. PROMs, patient‐reported outcome measures; SCTG, subepithelial connective tissue graft; VCMX, volume‐stable collagen matrix.

Patient demographics are presented in Table 1. A total of 20 patients were enrolled in the study and received soft tissue augmentation with either a volume‐stable collagen matrix (VCMX; *n* = 10) or a subepithelial connective tissue graft (SCTG; *n* = 10). At baseline, smoking exposure was low in both groups, as heavy smokers (> 10 cigarettes/day) were not eligible for inclusion. Over the course of the study, several patients became unavailable for continued monitoring due to emigration, death, COVID‐19‐related constraints, or withdrawal of consent. At the 7.5‐year follow‐up, 12 patients (seven in group VCMX; five in group SCTG) remained available for evaluation, as illustrated in the CONSORT flow diagram (Figure 1). Both implants and restorations remained fully functional, yielding a 100% survival rate over the 7.5‐year period.

**TABLE 1:** Patient demographics at baseline.

		Group SCTG	Group VCMX
Gender	*n* (female)	6	7
	*n* (male)	4	3
Age	Mean ± SD	43.4 ± 18.7	44.1 ± 12.8
	Median	47.5	46.0
	Q1; Q3	23.0; 60.0	39.0; 48.0
Cigarettes per day	Mean ± SD	1.0 ± 2.5	0.0 ± 0.0
	Median	0.0	0.0
	Q1; Q3	0.0; 0.0	0.0; 0.0

Abbreviations: Q1, first quartile; Q3, third quartile; SCTG, subepithelial connective tissue grafts; SD, standard deviation; VCMX, volume‐stable collagen matrix.

### 3.2 Mucosal Thickness (Primary Outcome)

**FIGURE 2:**
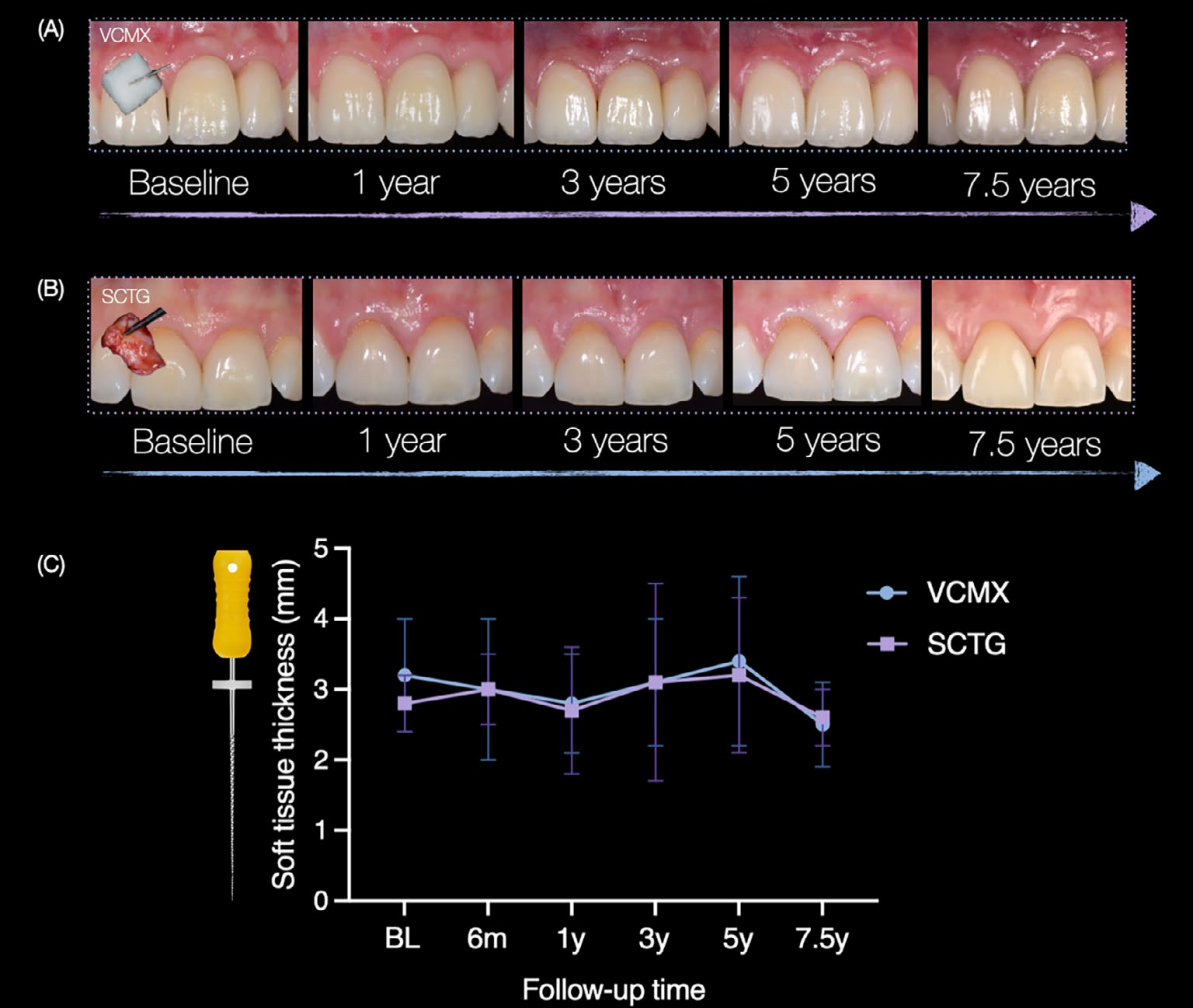
Representative cases in the volume‐stable collagen matrix (VCMX) group (A) and the subepithelial connective tissue graft (SCTG) group (B) at baseline and at 1, 3, 5, and 7.5 years. (C) Shows line plots of mean soft‐tissue thickness over time for both treatment groups across all follow‐up time points. Error bars indicate SD.

Mucosal thickness values at all time points are presented in Table 2, and longitudinal changes are illustrated in Figure 2. The mean mucosal thickness at baseline was 2.8 ± 0.4 mm in the SCTG group and 3.2 ± 0.7 mm in the VCMX group (adjusted mean difference; 0.7 [95% CI −0.1 to 1.1]; *p* = 0.088). Over time, mucosal thickness increased in both groups, with a statistically significant increase observed at 5 years compared with baseline (mean increase≈0.7 mm; *p* = 0.027), whereas changes at other time points were not significant. At the 7.5‐year follow‐up, mean mucosal thickness measured 2.6 ± 0.4 mm in the SCTG group and 2.5 ± 0.6 mm in the VCMX group (adjusted mean difference; −0.1 [95% CI −1.0 to 0.7]; *p* = 0.752). Linear mixed‐effects analysis showed no significant group‐by‐time interaction, indicating a similar pattern in both groups. Intergroup differences fluctuated slightly across follow‐up intervals but did not reach statistical significance at any time point or overall (*p* > 0.05).

**TABLE 2:** Linear mixed‐effects models were used for mucosal thickness, probing depth (PD), and marginal bone level (MBL), and generalized estimating equation models were used for plaque control record and bleeding on probing (BOP).

Parameter	Timepoint	Group SCTG		Group VCMX		Comparison	
		Mean (SD)	Median (Q1; Q3)	Mean (SD)	Median (Q1; Q3)	Adjusted mean treatment difference (95% CI)	*p*
Mucosal thickness (mm)	Baseline	2.8 (0.4)	3.0 (2.5; 3.0)	3.2 (0.8)	3.0 (3.0; 4.0)	0.7 (−0.1; 1.5)	0.088
	6 months FU	3.0 (0.5)	3.0 (3.0; 3.2)	3.0 (1.0)	3.0 (2.0; 4.0)	−0.0 (−0.8; 0.8)	0.944
	1 year FU	2.7 (0.9)	2.5 (2.0; 3.8)	2.8 (0.7)	3.0 (2.0; 3.0)	0.1 (−0.6; 0.9)	0.639
	3 years FU	3.1 (1.4)	3.0 (1.9; 4.2)	3.1 (0.9)	3.2 (2.6; 4.0)	0.1 (−0.7; 0.9)	0.799
	5 years FU	3.2 (1.1)	3.2 (3.0; 3.8)	3.4 (1.2)	3.0 (3.0; 4.0)	0.1 (−0.6; 0.9)	0.751
	7.5 years FU	2.6 (0.4)	2.7 (2.1; 3.0)	2.5 (0.6)	2.5 (2.0; 3.0)	−0.1 (−1.0; 0.7)	0.752
Plaque control record (%)	Baseline	0.1 (0.0)	0.1 (0.0; 0.1)	0.0 (0.0)	0.0 (0.0; 0.0)	−0.0 (−0.1; 0.0)	0.087
	6 months FU	0.2 (0.2)	0.1 (0.0; 0.3)	0.1 (0.2)	0.0 (0.0; 0.2)	−0.0 (−0.3; 0.1)	0.649
	1 year FU	0.0 (0.0)	0.0 (0.0; 0.0)	0.1 (0.2)	0.0 (0.0; 0.0)	0.0 (−0.1; 0.2)	0.412
	3 years FU	0.0 (0.0)	0.0 (0.0; 0.1)	0.1 (0.3)	0.0 (0.0; 0.0)	0.0 (−0.2; 0.2)	0.774
	5 years FU	0.1 (0.1)	0.2 (0.2; 0.2)	0.2 (0.2)	0.3 (0.0; 0.3)	0.1 (−0.2; 0.3)	0.295
	7.5 years FU	0.3 (0.5)	0.1 (0.0; 0.1)	0.2 (0.4)	0.0 (0.0; 0.0)	−0.1 (−0.6; 0.4)	0.674
Probing depth (mm)	Baseline	3.0 (0.3)	3.0 (3.0; 3.2)	3.1 (0.4)	3.3 (3.2; 3.3)	−0.0 (−0.5; 0.4)	0.814
	6 months FU	2.9 (0.4)	2.8 (2.7; 3.2)	3.2 (0.3)	3.2 (3.2; 3.4)	0.3 (−0.1; 0.8)	0.186
	1 year FU	2.9 (0.7)	2.5 (2.5; 2.8)	2.6 (0.3)	2.7 (2.6; 2.7)	−0.3 (−0.8; 0.2)	0.263
	3 years FU	3.9 (0.5)	3.7 (3.7; 4.2)	3.4 (0.6)	3.7 (2.9; 3.7)	−0.5 (−1.1; −0.0)	0.030
	5 years FU	3.5 (0.6)	3.7 (3.3; 3.8)	3.3 (0.6)	3.3 (3.0; 3.6)	−0.1 (−0.7; 0.3)	0.484
	7.5 years FU	3.3 (0.5)	3.5 (3.0; 3.7)	3.2 (0.5)	3.0 (2.9; 3.5)	−0.1 (−0.6; 0.4)	0.683
Beeding on probing (%)	Baseline	0.1 (0.1)	0.0 (0.0; 0.2)	0.1 (0.2)	0.1 (0.0; 0.3)	0.0 (−0.1; 0.2)	0.388
	6 months FU	0.2 (0.2)	0.2 (0.0; 0.5)	0.2 (0.3)	0.0 (0.0; 0.4)	−0.0 (−0.3; 0.2)	0.886
	1 year FU	0.0 (0.0)	0.0 (0.0; 0.0)	0.2 (0.4)	0.0 (0.0; 0.2)	0.2 (−0.0; 0.4)	0.136
	3 years FU	0.2 (0.1)	0.3 (0.1; 0.3)	0.3 (0.3)	0.2 (0.0; 0.5)	−0.0 (−0.3; 0.3)	0.990
	5 years FU	0.0 (0.1)	0.0 (0.0; 0.0)	0.3 (0.2)	0.2 (0.2; 0.5)	0.2 (0.0; 0.5)	0.009
	7.5 years FU	0.3 (0.3)	0.3 (0.2; 0.7)	0.5 (0.3)	0.5 (0.3; 0.7)	0.0 (−0.2; 0.4)	0.617
Marginal bone level (mm)	Baseline	−0.2 (0.4)	−0.1 (−0.3; 0.2)	0.2 (0.7)	0.3 (0.1; 0.5)	0.4 (−0.2; 1.1)	0.172
	3 years FU	−0.5 (0.5)	−0.4 (−0.6; −0.3)	−0.5 (1.0)	−0.4 (−0.4; −0.0)	−0.0 (−0.7; 0.6)	0.972
	5 years FU	−0.6 (0.5)	−0.5 (−0.6; −0.3)	−0.4 (1.1)	−0.1 (−0.3; 0.0)	0.2 (−0.5; 0.9)	0.557
	7.5 years FU	−0.9 (1.1)	−0.3 (−1.3; −0.1)	−0.6 (1.0)	−0.4 (−0.5; −0.1)	0.1 (−0.5; 0.9)	0.656

*Note:* Models compared treatment groups over time and were adjusted for treatment group, time, and their interaction. Effect sizes are reported as adjusted mean differences with 95% CIs.

Abbreviations: CI, confidence interval; FU, follow‐up; Q1, first quartile; Q3, third quartile; SCTG, subepithelial connective tissue grafts; SD, standard deviation; VCMX, volume‐stable collagen matrix.

### 3.3 Buccal Contour Changes of the Peri‐Implant Tissues

**FIGURE 3:**
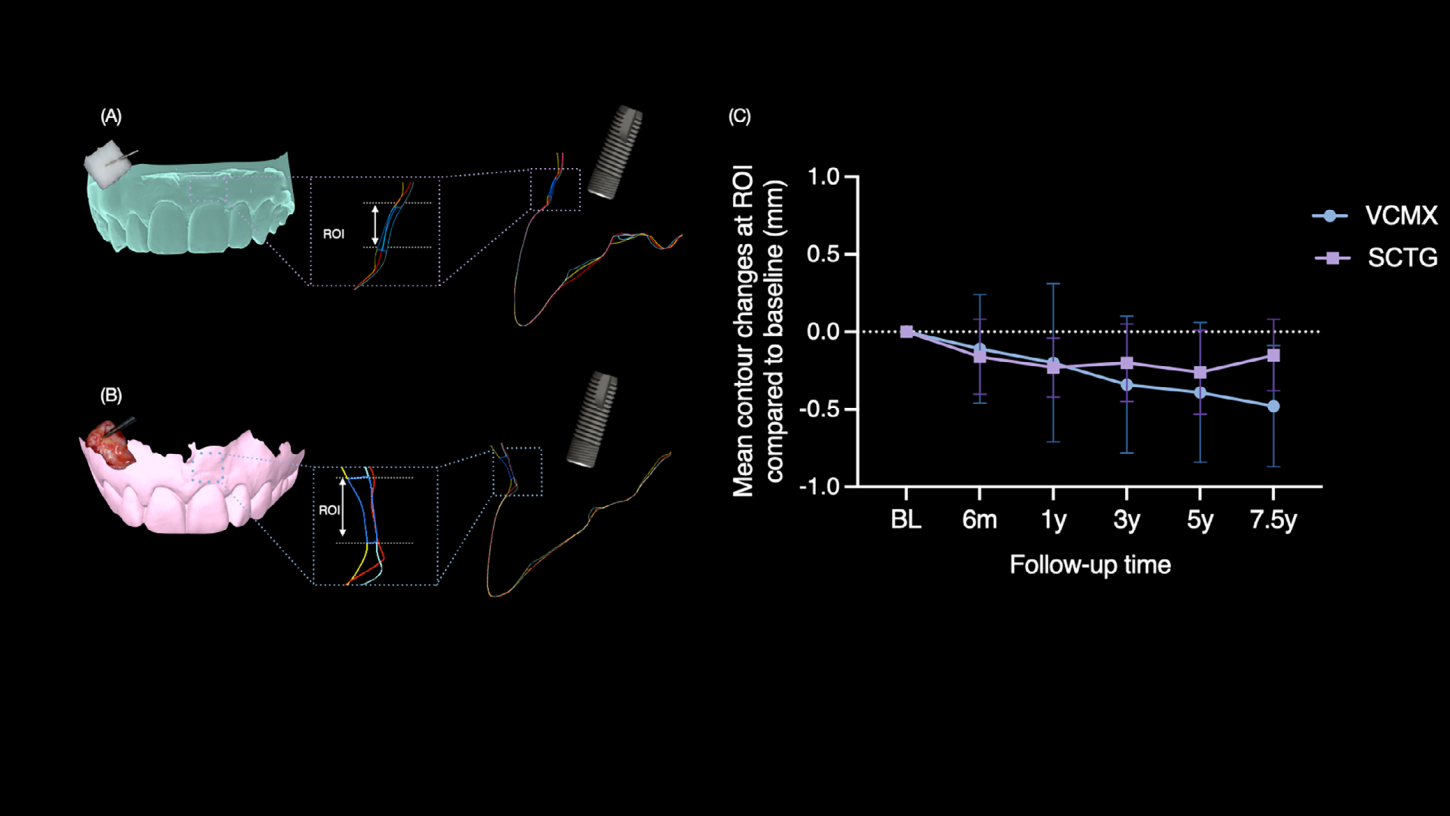
Digital workflow for the assessment of buccal contour changes based on the superimposition of serial intraoral scans in the VCMX (A) and SCTG (B) groups. (C) Shows line plots of mean changes in buccal contour position relative to baseline within the region of interest over time for both treatment groups. Error bars indicate SD.

At the 7.5‐year evaluation, volumetric analysis revealed minimal contour reduction in the SCTG group (−0.1 ± 0.2 mm), whereas the VCMX group demonstrated a greater decrease (−0.4 ± 0.4 mm), corresponding to an adjusted mean difference of −0.2 mm (95% CI −0.5 to 0.0; *p* = 0.068). Over time, mixed‐effects modeling showed no meaningful differences between groups at baseline or early follow‐up. At later intervals, however, the differences between SCTG and VCMX progressively increased, indicating greater volumetric shrinkage in the VCMX group. Although individual contrasts did not reach statistical significance, the overall pattern suggests a consistent trend toward increased long‐term contraction in the VCMX group. All contour changes across timepoints are reported in Table 3 and visually represented in Figure 3.

**TABLE 3:** Profilometric contour changes within each treatment group compared with baseline across time points and between group differences over time.

Timepoint	Group SCTG		Group VCMX		Comparison	
	Mean (SD)	Median (Q1, Q3)	Mean (SD)	Median (Q1, Q3)	Adjusted mean treatment difference (95% CI)	*p*
Contour changes						
ΔBL—6 months	−0.1 (0.2)	−0.1 (− 0.3; 0)	−0.1 (0.3)	0.0 (−0.2; 0.1)	0.0 (−0.2; 0.3)	0.751
ΔBL—1 year	−0.2 (0.1)	−0.2 (−0.3; −0.1)	−0.2 (0.5)	−0.1 (−0.3; 0.1)	0.0 (−0.2; 0.3)	0.846
ΔBL—3 years	−0.2 (0.2)	−0.1 (−0.3; 0.1)	−0.3 (0.4)	−0.2 (−0.7; −0.0)	−0.1 (−0.4; 0.1)	0.372
ΔBL—5 years	−0.2 (0.2)	−0.3 (−0.4; −0.1)	−0.3 (0.4)	−0.3 (−0.9; −0.1)	−0.0 (−0.3; 0.1)	0.515
ΔBL—7.5 years	−0.1 (0.2)	−0.2 (−0.3; −0.1)	−0.4 (0.4)	−0.3 (−0.9; −0.2)	−0.2 (−0.5; 0.0)	0.068

*Note:* Linear mixed‐effects models were used and adjusted for treatment group, time, and their interaction. Effect sizes are reported as adjusted mean differences with 95% CIs.

Abbreviations: BL, baseline; CI, confidence interval; Q1, first quartile; Q3, third quartile; SCTG, subepithelial connective tissue grafts; SD, standard deviation; VCMX, volume‐stable collagen matrix.

### 3.4 Clinical Outcomes

Clinical parameters are summarized in Table 2. In general, no statistically significant differences were found between groups for plaque levels, bleeding on probing, or probing depth at any timepoint. For BoP, a statistically significant between‐group difference was only observed at the 5‐year follow‐up, with higher values in the SCTG group compared with the VCMX group. No cases of peri‐implantitis were identified in either VCMX or SCTG sites. Peri‐implant mucositis was observed in fewer than 30% of patients in both groups, while all remaining sites exhibited a healthy peri‐implant status.

### 3.5 Radiographic Results

Radiographic outcomes (MBLs) are reported in Table 2. At 7.5 years, mean marginal bone levels were −0.90 ± 1.10 mm in the SCTG group and −0.65 ± 0.99 mm in the VCMX group, with no significant difference between treatments (adjusted mean difference 0.1 mm [95% CI −0.5 to 0.9], *p* = 0.656). Marginal bone levels remained stable over time in both groups, with no statistically significant differences at any time point.

### 3.6 Esthetic Outcomes

All esthetic outcomes are presented in Table 4. At crown delivery (BL), mean PES values were 8.6 ± 4.0 in the SCTG group and 9.4 ± 1.1, with no significant differences (adjusted mean difference 0.8 [95% CI −2.0 to 3.7]; *p* = 0.578). At 7.5 years of follow‐up, the PES remained stable in both groups, measuring 9.2 ± 3.3 in SCTG and 9.1 ± 3.2 again with no significant differences between them (adjusted mean difference ‐ 0.4 [95% CI −3.4 to 2.4]; *p* = 0.744). Within‐group changes over time showed a general trend toward improvement in both treatment groups. No significant group‐by‐time interactions were detected, indicating that the trajectory of PES improvement over time was similar between VCMX and SCTG. The mean mid‐facial recession was 0.6 ± 0.6 mm in the VCMX group, whereas the SCTG group showed a mean gain of 0.1 ± 0.7 mm. Collectively, these findings suggest that VCMX and SCTG may provide comparable long‐term esthetic outcomes.

**TABLE 4:** Pink esthetic scores (PES) in each treatment group across the different timepoints.

PES	Timepoint	Group SCTG		Group VCMX		Comparison	
		Mean (SD)	Median (Q1; Q3)	Mean (SD)	Median (Q1; Q3)	Adjusted mean treatment difference (95% CI)	*p*
Esthetic outcomes (PES)							
	Crown delivery	8.6 (4.0)	10 (6; 11)	9.4 (1.1)	9 (9; 10)	0.8 (−2.0; 3.7)	0.578
	6‐month FU	10.4 (3.0)	10 (10; 12)	8.7 (2.2)	8.5 (7.25; 9.75)	−1.8 (−4.8; 1.1)	0.224
	1 year FU	9.2 (2.4)	9 (8; 11)	8.9 (2.8)	8 (8; 11)	−0.3 (−3.2; 2.5)	0.818
	3 year FU	9.6 (2.5)	10 (9; 10)	9.0 (2.8)	9 (6.5; 11)	−0.6 (−3.5; 2.3)	0.687
	5 year FU	9.6 (3.0)	11 (8; 12)	10.1 (2.8)	10.5 (8.5; 12.5)	0.1 (−2.7; 3.1)	0.910
	7.5 year FU	9.2 (3.3)	11 (8; 11)	9.1 (3.2)	11 (7; 11)	−0.4 (−3.4; 2.4)	0.744

*Note:* Linear mixed‐effects models were used and adjusted for treatment group, time and their interaction. Effect sizes are reported as adjusted mean differences with 95% CIs.

Abbreviations: CI, confidence interval; FU, follow‐up; Q1, first quartile; Q3, third quartile; SCTG, subepithelial connective tissue grafts; SD, standard deviation; VCMX, volume‐stable collagen matrix.

### 3.7 Patient‐Reported Outcome Measures

OHIP‐14 scores did not differ between groups at baseline (*p* = 0.536), and neither group showed significant changes over time. No group‐by‐time interactions were detected and post hoc analyses confirmed no between‐group differences at any timepoint (all *p* > 0.05). The overall joint test was nonsignificant (*p* = 0.135), indicating that VCMX and SCTG demonstrated comparable oral‐health related quality of life PROs throughout follow‐up (Table 5).

**TABLE 5:** Oral Health Impact Profile‐14 (OHIP‐14) in each treatment group across the different timepoints.

OHIP‐14	Timepoint	Group SCTG		Group VCMX		Comparison	
		Mean (SD)	Median (Q1; Q3)	Mean (SD)	Median (Q1; Q3)	Adjusted mean treatment difference (95% CI)	*p*
Patient‐reported outcomes							
	Crown delivery	1.0 (1.7)	0 (0; 1)	2.4 (6.0)	0 (0; 0.5)	1.4 (−3.0; 5.9)	0.536
	6‐month FU	0.8 (1.8)	0 (0; 0)	1.7 (3.6)	0 (0; 0.75)	0.7 (−2.2; 3.7)	0.619
	1 year FU	1.0 (2.2)	0 (0; 0)	1.1 (3.0)	0 (0; 0)	0.1 (−2.6; 2.9)	0.921
	3 year FU	0.0 (0.0)	0 (0; 0)	0.6 (1.1)	0 (0; 0.5)	0.5 (−0.2; 1.3)	0.168
	5 year FU	1.0 (1.4)	0 (0; 2)	0.8 (0.4)	0 (0; 0)	−0.9 (−2.2; 0.2)	0.130
	7.5 year FU	3.2 (7.2)	0 (0; 0)	0.7 (0.9)	0 (0; 0.75)	−2.4 (−8.3; 3.4)	0.409

*Note:* Generalized Estimating Equations (GEE) models were used and adjusted for treatment group, time, and their interaction. Effect sizes are reported as adjusted mean differences with 95% CIs.

Abbreviations: CI, confidence interval; FU, follow‐up; Q1, first quartile; Q3, third quartile; SCTG, subepithelial connective tissue grafts; SD, standard deviation; VCMX, volume‐stable collagen matrix.

## 4 Discussion

The present long‐term follow‐up of a randomized clinical trial compared a volume‐stable collagen matrix (VCMX) and a subepithelial connective tissue graft (SCTG) for peri‐implant mucosal thickening in the esthetic zone. The findings predominantly revealed:

- No clinically meaningful long‐term differences in mucosal thickness or buccal contours between SCTG and VCMX
- Stable clinical and radiographic parameters in both groups over the entire 7.5‐year follow‐up
- Similar esthetic and PROs, regardless of the augmentation material

### 4.1 Long‐Term Maintenance of Mucosal Thickness

Evidence on the long‐term behavior of augmented peri‐implant mucosa using soft tissue substitutes remains limited. Most published studies focus on outcomes within 1–3 years [15, 22, 30, 31, 32, 33, 38]. Data extending beyond 5 years are rare [29], and even fewer studies have assessed collagen matrices over such prolonged intervals. In this context, the present study provides valuable longitudinal information. Similar patterns were observed in a recent multicenter RCT with 3‐year follow‐up, where mucosal thickness decreased from 3.9 ± 1.4 mm to 2.6 ± 1.1 mm in the VCMX group and from 3.8 ± 1.3 mm to 2.9 ± 1.2 mm in the SCTG group, resulting in an estimated intergroup difference of only 0.2 mm (*p* = 0.587) [39]. Although the follow‐up in that study was shorter, the magnitude and direction of the changes aligned closely with the modest reductions observed in the present long‐term evaluation. The stability demonstrated in the current trial may be attributable to several factors, including meticulous surgical execution, consistent operator expertise and the natural maturation of augmented tissues following the initial remodeling phase. Collectively, these elements likely contributed to predictable and harmonious outcomes in both groups, despite the use of different grafting materials.

### 4.2 Digital Volumetric Analysis and Contour Stability

Beyond linear mucosal thickness measurements obtained with an endodontic file, STL‐based volumetric analysis provides a more comprehensive assessment of peri‐implant soft‐tissue behavior [32, 33, 40]. Both treatment groups demonstrated minimal contour contraction over 7.5 years. Although VCMX showed slightly greater shrinkage (adjusted mean difference: 0.28 mm), this difference was not clinically perceptible. Notably, a similar difference of approximately 0.3 mm was reported in a multicentre RCT evaluating the same grafting materials at 3 years, suggesting consistency across studies and follow‐up periods [31]. To our knowledge, no RCTs with more than 5 years of follow‐up are available, limiting direct comparison with the present long‐term outcomes. Nonetheless, the ≈0.3 mm difference observed here aligns with a recently commissioned systematic review and meta‐analysis, which reported a mean difference of 0.27 mm in favor of SCTG across five studies [41]. However, the included trials had only 1 year of follow‐up, considerably shorter than in the present investigation [41].

The long‐term stability observed in this study supports the concept that, after the initial remodeling phase [42], peri‐implant soft tissues reach a plateau of dimensional stability when sufficient mucosal thickness is established [43]. The high agreement between analogue and digital measurements further reinforces the conclusion that both SCTG and VCMX can maintain stable peri‐implant contours over time. A recent review also demonstrated that digital measurements of peri‐implant soft tissues are more accurate than conventional clinical assessments, enabling clinicians to more reliably diagnose, plan, and monitor peri‐implant soft‐tissue conditions and esthetics [40].

### 4.3 Clinical and Radiographic Stability

Clinical parameters, including probing depth, plaque accumulation and bleeding on probing, remained stable in both groups, following nearly identical trajectories throughout the follow‐up period. Peri‐implant mucositis occurred only sporadically and no cases of peri‐implantitis were observed. These findings indicate that neither augmentation approach compromises peri‐implant health and that both can maintain peri‐implant health when regular maintenance care is provided [44]. Similar short‐term stability has been documented in a multicenter 12‐month RCT comparing SCTG and VCMX, where both materials performed comparably across all clinical outcomes. In that study, probing depths remained within a narrow range (approximately 2.6–3.0 mm), bleeding on probing was consistently low (around 6%–7%), and radiographic bone levels changed minimally from baseline to 12 months (SCTG: 1.10–0.47 mm; VCMX: 0.94–0.43 mm), with no significant between‐group differences [30].

The radiographic stability observed in the present study aligns with previous evidence suggesting that soft‐tissue augmentation, regardless of the material used, may contribute to the maintenance of peri‐implant health, potentially by enhancing the soft‐tissue phenotype and reinforcing the epithelial seal [43].

### 4.4 Esthetic and PROs

Esthetic outcomes, assessed using the PES, remained consistently high and stable over 7.5 years, with an adjusted mean difference of only 0.4 points between groups (Table 4). Neither augmentation strategy showed superiority. This lack of detectable difference aligns with a 3‐year RCT reporting nearly identical PES values between SCTG and VCMX (mean difference≈0.4 points; 10.5 for SCTG vs. 10.9 for VCMX) [31]. Historically, early esthetic advantages have been attributed to SCTG, however, these advantages may diminish or become clinically indistinguishable over time. Recent systematic reviews with meta‐analyses and accompanying editorials have similarly noted that clinicians may perceive a slight esthetic advantage with autogenous grafts, but these differences are typically too small to be appreciated by patients [41, 44, 45].

PROs mirrored the clinical and esthetic results. OHIP scores remained low across all time points, indicating minimal functional or psychosocial burden and high sustained satisfaction. Alternatively, the absence of PROM differences may relate to the limited sensitivity of currently available PROM instruments, which may not detect subtle changes in soft‐tissue contour or volume [21, 44, 46]. This underscores the importance of integrating both clinical assessments and patient perspectives when evaluating treatment outcomes.

### 4.5 Strengths and Limitations

A key strength of this study is its extended follow‐up period, which is uncommon in soft‐tissue augmentation research and provides rare insight into the long‐term behavior of augmented peri‐implant tissues at single‐tooth implants in the esthetic zone. The use of both analogue and digital measures also strengthens the reliability of the findings [40]. Several limitations should also be acknowledged. First, the sample size was small, which may have introduced bias, and the high dropout rate further reduced the statistical power of the long‐term follow‐up. This limited the ability to detect clinically meaningful or statistically significant between‐group differences and likely explains the absence of observed differences between the groups. Second, minor anatomical changes over time (e.g., recession) may have influenced the accuracy of the analysis. Because the ROI was fixed at baseline, it may not have fully corresponded to the intended tissue zone at follow‐up, potentially leading to an underestimation of the true volume loss. For future studies, analyses could be refined by reporting coronal, middle and apical zones separately, using standardized and stable reference points. Third, the hybrid (indirect) workflow used to obtain the STL files, which may introduce inherent inaccuracies due to additional processing steps and affect the volumetric analyses. Fourth, preaugmentation mucosal thickness was not formally assessed prior to grafting, as the present investigation represents a long‐term follow‐up of a previously conducted randomized clinical trial. Consequently, baseline measurements reflect tissue conditions at crown delivery following the initial intervention rather than the original gingival phenotype. Finally, measurements performed with endodontic files may be prone to error, including instability of the rubber stop, which can move along the instrument shaft and inherent rounding errors [47].

## 5 Conclusions

Both augmentation approaches were associated with stable peri‐implant mucosal dimensions, preserved marginal bone levels, and favorable esthetic and PROs over a 7.5‐year period in implant sites presenting at least 2 mm mucosal thickness at crown delivery following a previous soft tissue procedure. While volume‐stable collagen matrices might be a viable alternative to SCTG for peri‐implant soft‐tissue augmentation, adequately powered randomized controlled trials are needed to confirm these observations.

## Author Contributions

All authors made substantial contributions to this study. D.S.T. and R.E.J. contributed to the conception and design of the study. F.J.S., M.G.L., N.N., T.J.W.G. contributed to the clinical phases of the study and collected the data. F.J.S. interpreted the data and performed the statistical analysis. F.J.S. and M.G.L. drafted the manuscript and R.E.J. and D.S.T. critically reviewed and revised it.

## Funding

The original Randomized Controlled Trial was funded by Geistlich Pharma AG, Wolhusen, Switzerland and by the Clinic of Reconstructive Dentistry, University of Zurich, Zurich, Switzerland.

## Conflicts of Interest

The authors declare no conflicts of interest.

## Data Availability

The data that support the findings of this study are available from the corresponding author upon reasonable request.
